# Analysis of Ubiquitin-Conjugating Enzyme E2T (UBE2T) Protein Levels in the Bone Marrow Biopsy Specimens of Patients With Multiple Myeloma

**DOI:** 10.7759/cureus.82571

**Published:** 2025-04-19

**Authors:** Fei Wang, Na Yu, Rong Xia

**Affiliations:** 1 Internal Medicine, Huashan Hospital, Fudan University, Shanghai, CHN; 2 Pathology, Huadong Hospital, Fudan University, Shanghai, CHN

**Keywords:** beta-2-microglobulin, bone marrow biopsy, lactate dehydrogenase, multiple myeloma, protein expression, ubiquitin conjugating enzyme e2 t

## Abstract

Introduction

Several studies have reported that the ubiquitin-conjugating enzyme E2 T (UBE2T) is overexpressed in multiple myeloma (MM). This has been assessed via bioinformatic analysis, which represents mRNA expression. However, biological experimental evidence is lacking. Moreover, the protein levels of UBE2T in tissue obtained via bone marrow biopsy are not clear. To identify newer potential therapeutic targets for MM, this study aimed to assess the expression of the UBE2T protein by immunohistochemical (IHC) staining of bone marrow biopsy specimens and also collected clinical data for a correlation analysis.

Methods

Bone marrow biopsy specimens were obtained from the pathology department between December 2022 and December 2024. The expression of UBE2T protein in them was evaluated by IHC staining. Clinical data of the patients were also collected. These included gender, age, Revised International Staging System (R-ISS) staging category, overall survival and progression-free survival time, and concentrations of hemoglobin (Hb), creatinine (Cr), calcium (Ca), serum beta-2-microglobulin (β2-MG), serum albumin (ALB), and serum lactate dehydrogenase (LDH). Furthermore, we investigated the expression of UBE2T in patients with MM belonging to different R-ISS staging categories and its relationship with Hb, Cr, Ca, ALB, β2-MG, and LDH concentrations. Finally, a survival analysis was performed.

Results

Bone marrow biopsy specimens were obtained from 77 patients with MM and 16 patients with non-hematological conditions (control group). The UBE2T protein was weakly expressed in the control group (IHC results were negative or weakly positive), while it was significantly greater in specimens obtained from patients with MM (P< 0.001 vs. control). The patients with MM were further divided into three groups according to their clinical Revised International Staging System (R-ISS) staging. Compared to patients in R-ISS stage I, the UBE2T protein expression was significantly increased in those in stage II (P<0.05) and III (P<0.0001), whereas patients in stage III showed significantly higher levels than patients in stage II (P<0.01). Additionally, the level of UBE2T protein expression was positively correlated with the serum concentrations of β2-MG (P < 0.001, R^2^=0.211) and LDH (P < 0.001, R^2^=0.192). Further, the one-year progression-free survival rate was significantly higher in the low-expression (87.18%, 34/39) vs. the high-expression group (71.05%, 27/38; P<0.05).

Conclusions

The UBE2T protein is highly expressed in bone marrow biopsy specimens from patients with MM and positively correlates with R-ISS staging categories and serum concentrations of β2-MG and LDH. The comparative one-year progression-free survival rate was also significantly higher in the low-expression vs. the high-expression group. Although larger scale and longer follow-up studies are needed, UBE2T may become a potential indicator for MM detected via bone marrow biopsy and a novel target for its therapy.

## Introduction

Multiple myeloma (MM) is a malignant hematological disease originating from the plasma cells within the bone marrow. It is the second most common hematological malignancy. Its main clinical manifestations are calcium elevation, renal insufficiency, anemia, and bone destruction [1]. Recently, with the clinical application of new drugs such as proteasome inhibitors, immunomodulators, and monoclonal antibodies, the survival of patients with MM has improved significantly [2,3]. However, MM is still incurable, and shows significant heterogeneity in terms of clinical manifestations, genomic features, therapeutic efficacy, etc. [4]. Therefore, the exploration of new biomarkers for MM and the study of individualized treatment plans remain key areas of focus for research. 

The ubiquitin proteasome system (UPS) consists of ubiquitin, ubiquitinating and deubiquitinating enzymes, and proteasomes [5]. Among these, ubiquitinating enzymes include the E1-activating enzymes, E2-binding enzymes, and E3 ligases. The primary function and mechanism of the UPS is to regulate protein degradation by ubiquitination of the target proteins to maintain protein homeostasis and prevent aggregation of misfolded proteins. Notably, myeloma cells produce large amounts of immunoglobulins and, therefore, rely heavily on the UPS system to maintain stable cellular function [6]. The key mechanism of immunomodulators used in myeloma therapy is to promote the ubiquitination of transcription factors IKAROS Family Zinc Finger 1 (IKZF1) and IKAROS Family Zinc Finger 3 (IKZF3) through the action of cereblon, an E3 ligase. As such, this activates T cells, NK cells, and other immune effector cells, killing MM cells and exerting anti-myeloma effects [7]. Immunomodulators approved by the US Food and Drug Administration (FDA) include thalidomide, lenalidomide, and pomalidomide. On the other hand, proteasomes also play crucial role in maintaining the functional stability of myeloma cells. Proteasome inhibitors approved by the FDA for the treatment of MM include bortezomib, carfilzomib, and ixazomib. However, resistance to these inhibitors persists and is the leading cause of relapse and disease progression in patients [8]. In summary, UPS plays a crucial role in the survival of myeloma cells. Apart from cereblon and the proteasome, UPS includes many E1-activating, E2-binding, and deubiquitylating enzymes, and other E3 ligases. It has been reported that ubiquitin-conjugating enzyme E2T (UBE2T), an E2-binding enzyme, correlates with survival and prognosis in MM. This has been confirmed by bioinformatic analysis [9,10], which represents its mRNA expression. However, the protein levels of UBE2T in bone marrow biopsy specimens from patients with MM are not clear.

This study aimed to analyze the protein expression of UBE2T in such bone marrow biopsy specimens and to investigate its correlation with clinical indicators, thereby providing a basis for understanding the pathology in MM.

## Materials and methods

Source of clinical specimens and data collection

Bone marrow biopsy specimens were obtained from the pathology department of the Huadong Hospital, Fudan University, from December 2022 to December 2024. The patients with MM included were from the hematology department, whereas the control group included attendees from the non-hematology departments. Clinical data, including gender, age, Revised International Staging System (R-ISS) staging categories, overall survival and progression-free survival time, and concentrations of hemoglobin (Hb), creatinine (Cr), calcium (Ca), serum albumin (ALB), serum beta-2-microglobulin (β2-MG), and serum lactate dehydrogenase (LDH), were collected. The study was approved by the Ethics Committee of Huadong Hospital, Fudan University (Approval number: 20240157). The clinical research process was conducted in accordance with the Declaration of Helsinki of the World Medical Association and other relevant regulations. 

Inclusion and exclusion criteria

MM Group

Patients with MM were included in the study and were diagnosed by clinicians, and the diagnostic criteria were in accordance with the diagnostic criteria of the National Comprehensive Cancer Network (NCCN) and the International Myeloma Working Group (IMWG) [11,12]. The patients were 18 years or older, male or female, and were diagnosed for the first time. Also, records of their R-ISS [13] staging categories were available, and the pathologist confirmed that their bone marrow biopsy specimens contained tissue with pathological changes related to MM. Informed consent was obtained via telephone or outpatient follow-up.

Patients were excluded if MM was present along with other diseases and if it was not a first-time diagnosis or the patient had already been diagnosed in another hospital. Other patients who were excluded were those who were pregnant or breastfeeding, those with unclear pathological diagnostic information, insufficient amount of remaining paraffin samples, a lack of clinical R-ISS staging records, and those with other conditions that the investigators deemed did not meet the inclusion criteria.

These criteria are summarized in Table 1.

**Table 1:** Inclusion and exclusion criteria of the MM group MM, multiple myeloma; R-ISS, Revised International Staging System.

Category	Criteria
Inclusion	Patients aged 18 years or older
	Male or female
	Diagnosed as MM for the first time
	Medical records of R-ISS staging category were available
	Bone marrow biopsy tissue with MM pathological changes
	Informed consent obtained via telephone or outpatient follow-up
Exclusion	Combined with other diseases
	Not a first-time diagnosis or already diagnosed in another hospital
	Insufficient amount of remaining paraffin samples
	Pregnant or breastfeeding
	Other conditions that the investigators deemed did not meet the inclusion criteria

Control Group

Patients included in this group were 18 years or older, male or female, and their bone marrow biopsy specimens were confirmed by the pathologist as being approximately normal or showing no noticeable pathological changes. Their informed consent was obtained via telephone or during outpatient follow-up.

Patients who were pregnant or breastfeeding, had insufficient remaining paraffin samples, and other conditions that the investigators thought did not meet the inclusion criteria, were excluded. 

The inclusion and exclusion criteria of the control group are summarized in Table 2.

**Table 2:** Inclusion and exclusion criteria of the control group

Category	Criteria
Inclusion	Aged 18 years or older
	Male or female
	From non-hematology departments
	Bone marrow biopsy specimens confirmed by the pathologist as being approximately normal or showing no noticeable pathological changes
	Informed consent obtained (via telephone or outpatient follow-up)
Exclusion	Pregnant or breastfeeding
	Insufficient remaining paraffin samples
	Other conditions that the investigators thought did not meet the inclusion criteria

Immunohistochemical (IHC) staining

We prepared xylene Ⅰ, xylene Ⅱ, anhydrous ethanol, 90% ethanol Ⅱ, 85% ethanol, and 75% ethanol, and soaked the prepared bone marrow biopsy tissue sections in sequence for 15, 15, five, five, and five minutes, and finally placed them in distilled water to wash for 10 min. We then placed them in a citrate buffer antigen repair solution (pH 6.0), to be boiled and heated for 29 min, cooled to room temperature, and then washed them three times for five minutes each using phosphate-buffered saline (PBS; pH about 7.4). We then prepared 3% hydrogen peroxide solution, placed each slice in the solution, incubated them at room temperature while avoiding light for 25 min, and then washed them with PBS (pH 7.4) three times for five minutes each. We added 3% bovine serum albumin (BSA) and incubated them for one hour, and added primary UBE2T antibody (AP10105-2-AP, Proteintech, Illinois, US). This antibody was validated for specificity with knockdown controls according to the instructions. Other studies have also used this antibody for performing western blot and IHC staining experiments [14-16]. We incubated the specimens at 4°C overnight, washed them with PBS three times for five minutes each, added a secondary antibody (horseradish peroxidase or HRP labeled), and incubated at room temperature for one hour, washed them with with PBS three times for five minutes each, added 3,3′-Diaminobenzidine (DAB) staining solution, and rinsed the slices with double-distilled water to terminate the staining. We then immersed the slices in hematoxylin staining solution, re-stained them for two minutes, and rinsed them with double-distilled water to terminate the staining. We then placed the slices sequentially in 75% alcohol for six minutes, 85% alcohol for six minutes, 90% anhydrous ethanol Ⅰ for six minutes, and 100% anhydrous ethanol for six minutes. The slices were then placed in xylene Ⅰ and II for 15 minutes each to achieve dehydration and transparency. Finally, the slices were removed, dried slightly, and sealed with drops of neutral gum. We then observed them under a microscope and captured the images. The UBE2T expression was evaluated by two independent pathologists blinded to the clinical data. The staining intensity and percentage of positive tumor cells were recorded. The score for the dark brown stain which was clearly visible was recorded as three, brown as two, light brown as one, and blue nuclei as zero. Histoscore or H-score for each sample was calculated. H-score was calculated as (percentage of weakly stained cells×1) + (percentage of moderately stained cells×2) + (percentage of strongly stained cells×3).

Statistical analysis

The IBM SPSS Statistics for Windows, Version 25 (Released 2017; IBM Corp., Armonk, New York, United States) was used for statistical analysis. The chi-square test was used to analyze categorical variables, provided that all expected cell frequencies were ≥5. If this assumption was not met, Fisher’s exact test was applied for 2×2 tables and likelihood ratio test was utilized for a three-level categorical outcome. Effect sizes were reported as Phi(φ) (for 2×2 table) or Cramer’s V (for 3×2 table). Numerical means ± standard deviations were used to assess continuous variables. Means between two groups were tested using an independent sample t-test for data that satisfied the normal distribution assumption. Data that did not meet this assumption were analyzed using the Mann-Whitney U-test. For R-ISS stage comparisons, one-way ANOVA was utilized with Tukey as the post-hoc test. Correlation was analyzed using the Pearson correlation test. The multivariate analyses were performed to adjust for confounding factor including gender, age, and R-ISS stage. The difference of survival analysis was tested by the log-rank test. A p-value of less than 0.05 was considered statistically significant.

## Results

The study included 77 patients with MM and 16 patients with non-hematological conditions (control group). The average age of patients in the MM group was 68.87±8.53 years, while it was 65.36±8.78 years in the control group. The MM group included patients in R-ISS stage I (n=13), II (n=32), and III (n=32), while the control group included patients with pericardial effusion (n=6), Behçet's disease (n=3), gastrointestinal bleeding (n=2), malnutrition (n=2), duodenal ulcer (n=2), and gastric ulcer (n=1). The baseline information and clinical hematologic indexes of the study subjects are summarized in Tables 3, 4.

**Table 3:** Baseline information and hematologic profile of the patients in both groups MM, multiple myeloma; df, degrees of freedom; P, Probability; Hb, hemoglobin; Cr, creatinine; Ca, calcium; β2-MG, serum beta-2-microglobulin; ALB, albumin; LDH, lactate dehydrogenase.

Variable	MM group	Control group	df	Phi(φ)	P
Number	77	16			
Gender			1	0.025	＞0.05
Male	39	8			
Female	38	8			
Age (years)	68.87±8.53	65.36±8.78			＞0.05
Hb (g/L)	95.93±22.34	127.45±12.43			＜0.001
Cr (µmol/L)	141.16±150.14	69.82±25.29			＜0.05
Ca (mmol/L)	2.40±0.33	2.17±0.10			＜0.05
ALB (g/L)	39.07±8.22	47.78±13.08			＜0.05
β2-MG (mg/L)	7.05±7.78	2.09±1.24			＜0.05
LDH (U/L)	201.82±106.59	159.79±37.44			＞0.05

**Table 4:** Baseline information and hematologic profile of patients with MM as per their R-ISS staging category R-ISS, Revised International Staging System; df, degrees of freedom; P, Probability; Hb, hemoglobin; Cr, creatinine; Ca, calcium; β2-MG, serum beta-2-microglobulin; ALB, albumin; LDH, lactate dehydrogenase.

Variable	R-ISS Ⅰ	R-ISS Ⅱ	R-ISS Ⅲ	df	Cramer’s V	P
Number	13	32	32			
Gender				2	0.083	＞0.05
Male	7 (9.10%)	15 (19.48%)	17 (22.08%)			
Female	6 (7.79%)	17 (22.08%)	15 (19.48%)			
Age (y)	56.23±7.26	69.75±6.08	73.125±5.88			＜0.001
Hb (g/L)	98.45±23.52	91.28±26.34	71.43±14.02			＞0.05
Cr (µmol/L)	114.52±110.80	127.83±185.53	171.55±168.43			＞0.05
Ca (mmol/L)	2.25±0.18	2.42±0.36	2.43±0.35			＞0.05
ALB (g/L)	43.55±8.92	37.06±3.29	35.49±6.73			＞0.05
β2-MG (mg/L)	2.24±0.80	4.35±3.28	10.43±9.65			＜0.01
LDH (U/L)	154.82±40.93	174.35±63.08	243.15±136.99			＜0.05

**Figure 1:**
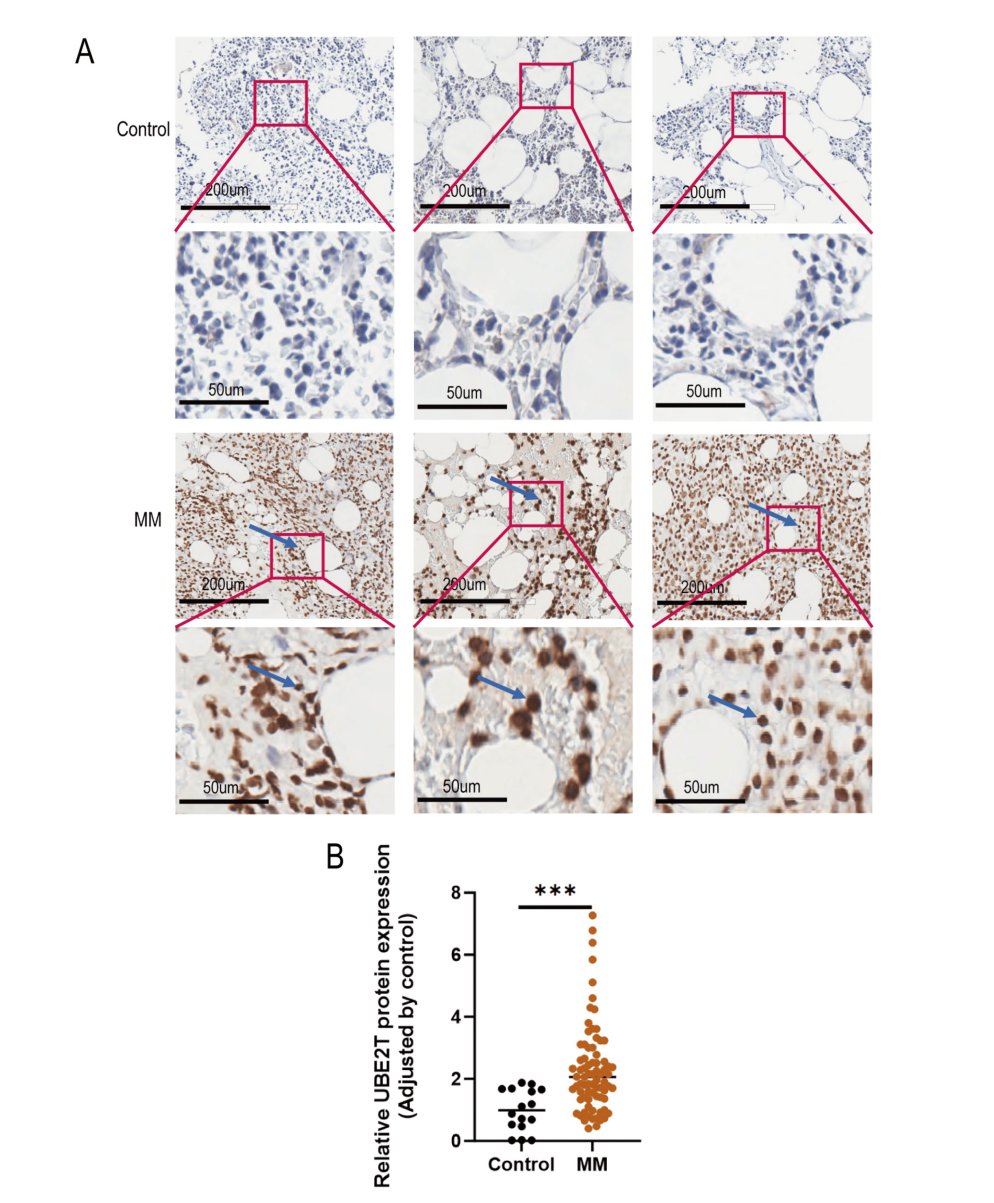
Increased UBE2T expression in bone marrow biopsy specimens obtained from patients with MM (A) Representative images (200×magnification) of UBE2T stain in bone marrow biopsy specimens from patients with MM (blue arrows) and the control group. (B) Comparison of the relative expression of UBE2T protein in specimens obtained from patients with MM (n=77) and the control group (n=16). ***P＜0.001; MM, multiple myeloma; UBE2T, ubiquitin-conjugating enzyme E2T.

The expression of UBE2T protein in the bone marrow biopsy tissue specimens from both the MM and control groups were examined by IHC experiments. The results showed that the UBE2T protein was weakly expressed in the control group (IHC results were negative or weakly positive). In contrast, its expression was significantly increased in patients with MM (P <0.001 vs. control) (Figure 1).

**Figure 2:**
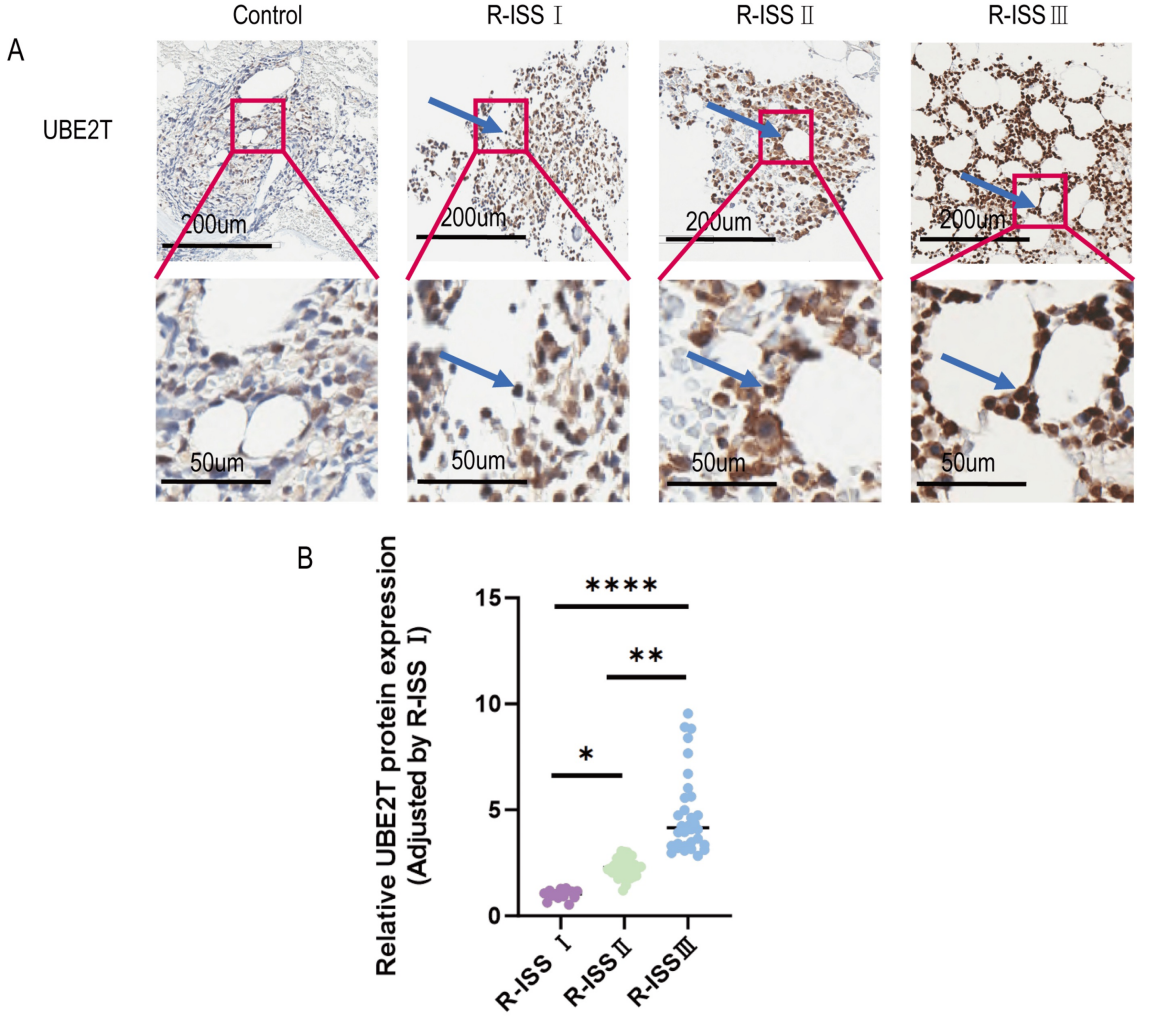
Expression of UBE2T protein in MM patients with different R-ISS stages (A) Representative images (200×magnification) of UBE2T staining in bone marrow biopsy specimens from patients at different R-ISS stages. (B) Statistical analysis of UBE2T protein expression in the different R-ISS staging groups. *P＜0.05; **P＜0.01; ****P＜0.0001; UBE2T, ubiquitin-conjugating enzyme E2T; R-ISS, Revised International Staging System.

Our study found that the UBE2T protein expression was significantly increased in R-ISS stage II (P<0.05) and III (P<0.0001) patients compared to stage I, while stage III patients showed significantly increased levels compared to those in stage II (P<0.01) (Figure 2).

**Figure 3:**
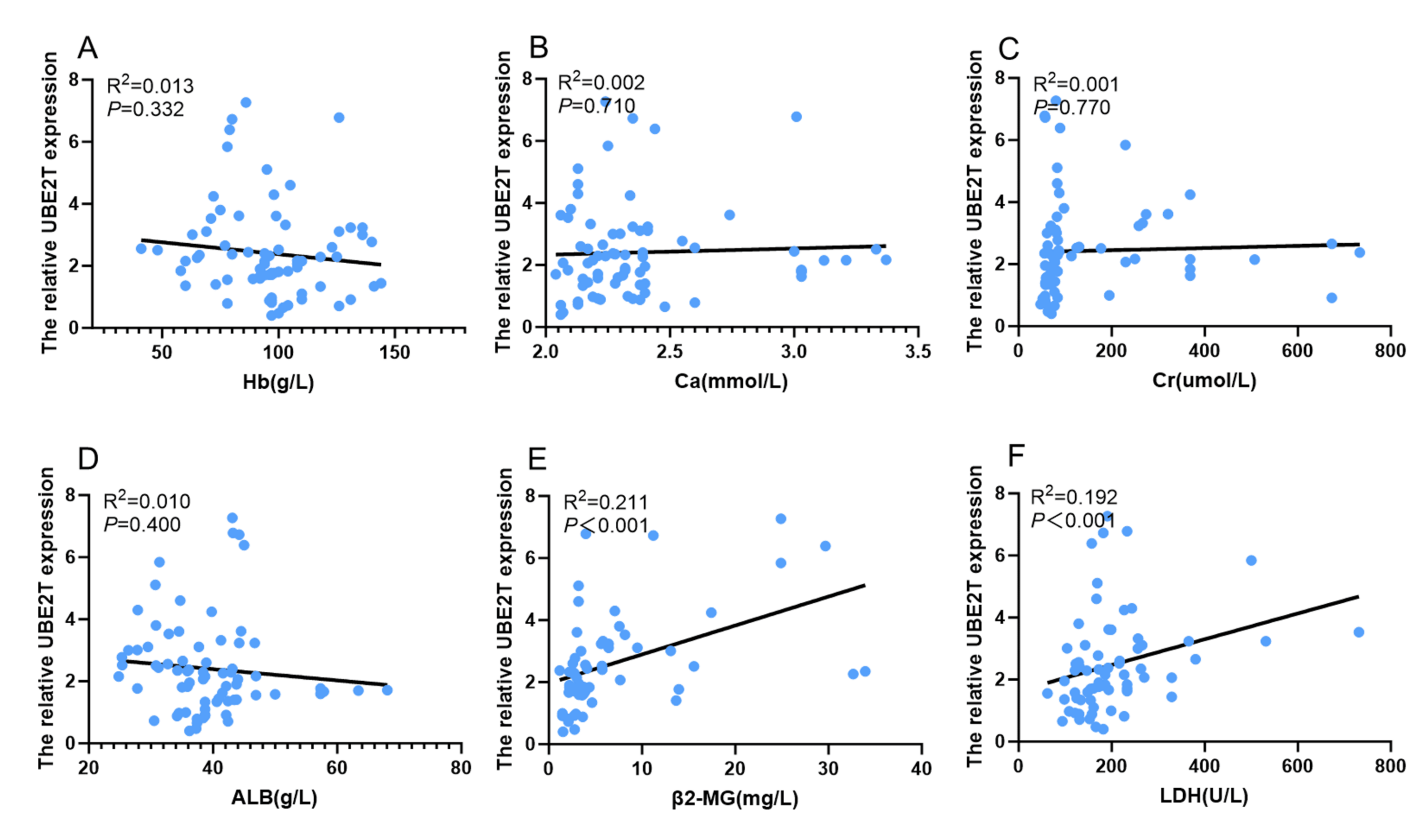
Correlation between UBE2T and clinical blood indices Analysis of the correlation between relative UBE2T protein expression levels and concentrations of (A) Hb, (B) Ca, (C) Cr, (D) ALB, (E) β2-MG, and (F) LDH. UBE2T, ubiquitin-conjugating enzyme E2T; Hb, hemoglobin; Cr, creatinine; Ca, calcium; β2-MG, serum beta-2-microglobulin; ALB, albumin; LDH, lactate dehydrogenase.

We further analyzed the correlation between the levels of UBE2T protein expression and clinical indicators, including the concentrations of Hb, Cr, Ca, ALB, and LDH, with the confounding factors being gender, age, and the R-ISS stage. The results showed that the UBE2T protein expression was positively correlated with the serum concentrations of β2-MG (P<0.001, R^2^=0.211) and LDH (P<0.001, R^2^=0.192) (Figure 3).

Since UBE2T is proposed as a potential prognostic marker in MM, we performed a survival analysis with several confounding factors including gender, age, and R-ISS stage. The patients were divided into two groups, using the median relative expression of UBE2T as the cut-off point. The one-year progression-free survival rate of the low-expression group (87.18%, 34/39) was significantly greater than the high-expression group (71.05%, 27/38; P＜0.05). On the other hand, though the one-year overall survival rate of the low-expression group (94.87%, 37/39) was greater than that of the high-expression group (89.47%, 34/38), the difference was not statistically significant (P＞0.05). This may be due to the relatively slow disease progression of MM and limited follow-up. These findings suggest that UBE2T may promote early progression but longer observational data (3-5 years) are required to gauge the impact on survival.

## Discussion

In this study, we found that UBE2T was highly expressed in the bone marrow biopsy specimens obtained from patients with MM. It was also correlated with R-ISS staging categories and the serum concentrations of β2-MG and LDH. Moreover, the one-year progression-free survival rate of the low-expression group was higher than that of the high-expression group. These results were consistent with the bioinformatic analyses that reported UBE2T mRNA levels were elevated in MM. UBE2T may serve as a novel indicator for bone marrow biopsy specimen testing in MM and can potentially be a new therapeutic target.

UBE2T, located at 1q32.1, is an E2 protease and belongs to the Fanconi anemia (FA) signaling pathway in the Kyoto Encyclopedia of Genes and Genomes (KEGG) signaling pathway. The FA pathway is necessary for the efficient repair of damaged DNA, which can help the body resist the endogenous and exogenous oncogenic aldehydes that can induce chromosome fragmentation [17-20]. Mutations in any of the genes in this pathway can cause FA. Consequently, FA is a genomic instability syndrome characterized by defective DNA repair, particularly in the repair of interstrand cross-links, which leads to chromosomal breakage. Importantly, individuals with FA face a significantly elevated cancer risk and are hundreds to thousands of times more likely than the general population to develop squamous cell carcinomas of the head and neck, esophagus, anus, and genital tract [21]. A critical component of this repair pathway is the FA complementation group D2 (FANCD2) protein. UBE2T binds to the ubiquitin ligase subunit of the FA core complex, known as FA complementation group L (FANCL), to mediate the monoubiquitination of FANCD2 [22]. Loss of UBE2T function, such as in UBE2T knockout cells, can result in DNA damage and the accumulation of abnormal chromosomes. Additionally, the FANCL proteins and inactive UBE2T are capable of auto-mono-ubiquitination [23]. This mechanism of self-deactivation plays a crucial role in the negative regulation of the FA signaling pathway.

In comparison, UBE2T expression is increased in many tumors. In pancreatic cancer, UBE2T reduces the effectiveness of gemcitabine by regulating pyrimidine metabolism and replication stress [24]. In hepatocellular carcinoma, UBE2T promotes carcinoma progression through ubiquitination of protein kinase B, and induces resistance to radiotherapy by ubiquitination of H2A histone family member X [25,26]. In gastric cancer, UBE2T promotes cancer progression through ubiquitination of receptor for activated C kinase 1 [27]. In glioblastoma, UBE2T promotes temozolomide drug resistance via wnt/β-catenin [28]. In triple-negative breast cancer, UBE2T promotes brain metastasis via the cell division control protein 42 (CDC42)/cluster of differentiation 276 (CD276) signaling axis [29], and targeting UBE2T suppresses breast cancer stem cells through chromobox protein 6 (CBX6)-mediated transcriptional repression [30]. As UBE2T has been reported to promote homologous recombination in MM [16], we hypothesized that it may have an impact on MM pathogenesis by affecting DNA replication, and thus protein synthesis and metabolism, including β2-MG or LDH. While this study focuses on clinical correlations, future work will explore the mechanistic roles of UBE2T in MM pathogenesis, particularly its impact on DNA replication and protein metabolism.

Some study limitations include the modest sample size (n=77) and relatively short follow-up period. Future multicenter studies with expanded cohorts and prolonged follow-up (≥3 years) are needed to validate the prognostic correlations and the potential therapeutic implications.

## Conclusions

The UBE2T protein is highly expressed in bone marrow biopsy tissue specimens obtained from patients with MM and positively correlates with the R-ISS staging categories, and serum concentrations of β2-MG and LDH. Moreover, compared to patients who had a high expression of the protein, the one-year progression-free survival rate was greater in those with a low expression. Although limitations such as the lack of longer follow-up and moderate sample size do exist, after considering previous studies, it is safe to conclude that UBE2T plays an important role in MM. This may guide further studies to evaluate UBE2T as a novel indicator for bone marrow biopsy testing and a new target for MM therapy. UBE2T can also be assessed along with other biomarkers to gauge the severity of MM.
